# The COVID-19 pandemic and heart failure: lessons from GUIDE-HF^[Author-notes ehac226-FM1]^

**DOI:** 10.1093/eurheartj/ehac226

**Published:** 2022-05-02

**Authors:** Martin R Cowie, John G F Cleland

**Affiliations:** Royal Brompton Hospital (Guy’s and St Thomas’ NHS Foundation Trust), London, UK; Institute of Health & Wellbeing, Robertson Centre for Biostatistics, University of Glasgow, Glasgow, UK; Department of Cardiology, Imperial College London, London, UK

## Abstract

**Graphical Abstract:**
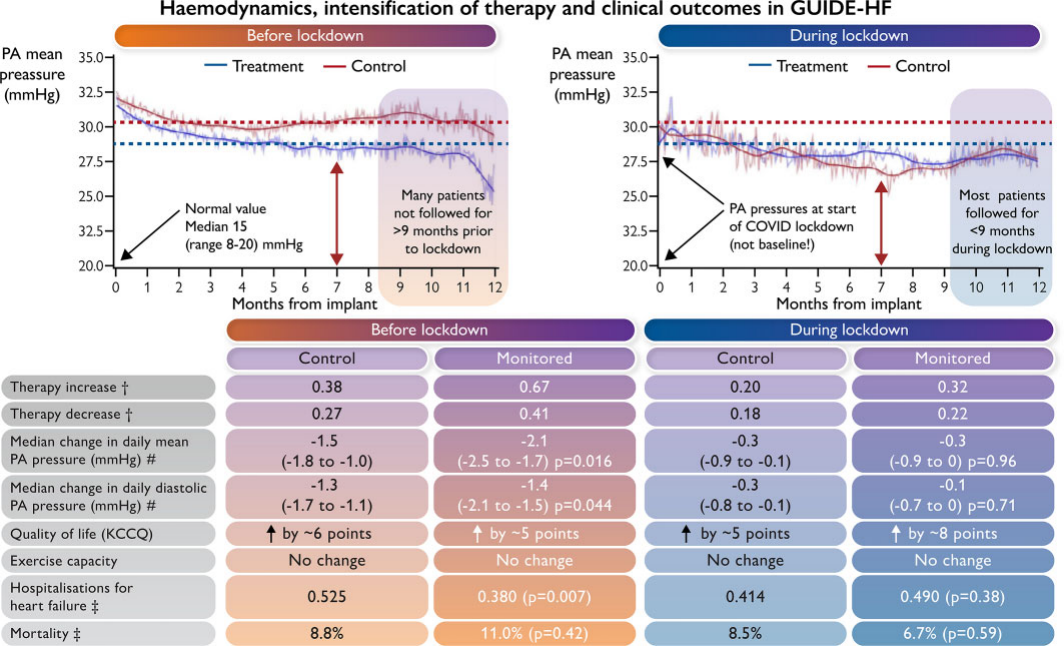
Average daily pressures before and during the COVID lockdown period. In the left-hand panel, only 40% of patients completed 12 months of monitoring prior to the COVID lockdown; many patients will have been censored between months 8 and 12. The right-hand panel shows pressures starting several months after trial inclusion; few patients will have been followed for more than 9 months. The red dotted line is an approximate average mean pulmonary artery pressure in the control group and the blue dotted line for the monitored group in the pre-COVID period. Note that even with monitoring, pulmonary artery pressures were far from normal. Rates of treatment intensification and impact on pulmonary artery pressures, quality of life, exercise capacity, hospitalizations, and death are shown numerically below the graphs. † = per patient per month; ‡ = annualised rate; # = daily average change in period; KCCQ = Kansas City Cardiomyopathy Questionnaire - improvements in scores from baseline are recalculated from Tables 1 and 6 from reference 6.

Average daily pressures before and during the COVID lockdown period. In the left-hand panel, only 40% of patients completed 12 months of monitoring prior to the COVID lockdown; many patients will have been censored between months 8 and 12. The right-hand panel shows pressures starting several months after trial inclusion; few patients will have been followed for more than 9 months. The red dotted line is an approximate average mean pulmonary artery pressure in the control group and the blue dotted line for the monitored group in the pre-COVID period. Note that even with monitoring, pulmonary artery pressures were far from normal. Rates of treatment intensification and impact on pulmonary artery pressures, quality of life, exercise capacity, hospitalizations, and death are shown numerically below the graphs. † = per patient per month; ‡ = annualised rate; # = daily average change in period; KCCQ = Kansas City Cardiomyopathy Questionnaire - improvements in scores from baseline are recalculated from Tables 1 and 6 from reference 6.

**This editorial refers to ‘The GUIDE-HF trial of pulmonary artery pressure monitoring in heart failure: impact of the COVID-19 pandemic’, by M.R. Zile *et al*., https://doi.org/10.1093/eurheartj/ehac114.**

The coronavirus disease 2019 (COVID-19) pandemic has led to major disruption in most aspects of human activity, including clinical trials. Normal social and professional interactions were severely limited, particularly during ‘lockdowns’. Attempting to mitigate the effects of the pandemic on clinical trials for conditions other than COVID-19, regulatory authorities, including the European Medicines Agency (EMA) and the Food & Drug Administration (FDA), issued guidance to trialists and sponsors that was designed to protect both participants and research staff and to maintain the scientific integrity of their efforts.^1–3^ Professional medical associations (including the European Society of Cardiology) also published consensus statements with suggestions on how to navigate through the problems that pandemics, such as COVID-19, pose to clinical trials.^4^

COVID-19, in addition to being a direct threat to participants, affects clinical trials in several other non-exclusive ways. Vulnerable patients, such as those with heart failure, were advised to avoid unnecessary contacts, including with research staff. This will have impeded trial enrolment, prevented many in-person visits, and increased withdrawal rates, unless the trial was designed or could be adapted to deliver remote management. Fear of COVID-19 infection will have increased efforts to prevent admissions by both clinical and research staff, and reduced the willingness of patients to be admitted. This might be expected to reduce hospitalizations but increase mortality.^5^ However, changes in patient behaviour, including the avoidance of respiratory infections other than COVID-19, might reduce both hospitalizations and mortality. The effects of pandemics on clinical trials are complex!

Some trials that had almost completed their planned follow-up decided to stop early, some temporarily suspended recruitment and/or follow-up, and others adapted their design to minimize in-person contacts and the risk of adverse events. However, many trials continued follow-up but altered their statistical analysis plans to include sensitivity analyses to investigate the impact of COVID-19, and associated lockdowns, on events. Pre-specifying such analyses adds scientific weight to the findings of trials but should be interpreted with caution as for all subgroup analyses.

One trial affected by the pandemic was GUIDE-HF, a multicentre randomized trial of pulmonary artery (PA) pressure monitoring for heart failure, which completed enrolment of 1022 participants in the USA and Canada on 20^th^ December 2019.^6–8^ The trial should have been relatively COVID resilient because management was designed to be delivered remotely. The primary endpoint was a composite of all-cause mortality and heart failure events, including hospitalizations and urgent attendance for intravenous diuretic therapy occurring within 12 months of randomization. The US national emergency declaration (‘lockdown’) occurred on March 13^th^ 2020, by which date most (72%) of the follow-up had occurred, although only 40% of participants had completed all 12 months. Overall, the trial narrowly missed its primary endpoint, which was mainly attributed to a decline in heart failure events in the control group during lockdown. Importantly, all participants were asked to take daily measurements of PA pressures but information was only disclosed to investigators in one arm of the trial; participants remained blinded as to whether or not the investigator was given the pressure readings. Thus, GUIDE-HF provides a unique opportunity to observe the haemodynamic effects of lockdown in patients with heart failure.^6^

Data collected at inclusion, completed before the pandemic struck, showed a median value for mean PA pressure of 31 mmHg (compared with ∼15 mmHg for healthy people) and for diastolic PA pressure 22 mmHg (∼12 mmHg for healthy people). During follow-up prior to lockdown, average daily mean PA pressure was reduced by only 2.1 mmHg in the monitored group compared with 1.7 mmHg in the control group (*P* = 0.016); for diastolic PA pressure, reductions were 1.4 mmHg and 1.3 mmHg (*P* = 0.044), respectively.

Despite the small effect on haemodynamics, a pre-specified analysis of outcome data acquired before lockdown suggested benefit, with a 19% reduction [95% confidence interval (CI) 0–34%, *P* = 0.049] in the relative risk of the composite endpoint, driven by a 24% relative and 15% absolute reduction in heart failure events (87% of events were hospitalizations rather than attendance for intravenous diuretic therapy). It is remarkable that such small differences in PA pressure, even cumulatively, could reduce the rate of worsening heart failure by such a substantial amount. Could a larger reduction in PA pressures exert a greater clinical benefit or would it cause an unacceptable increase in side effects? Unfortunately, the difference in PA pressures and in heart failure events were not sustained during lockdown, leading to a neutral overall result (12% relative risk reduction in the composite endpoint; *P* = 0.16). Fewer of those assigned to monitoring (5%) compared with the control group (9%) withdrew from the trial (*P* = 0.039) and rates did not increase during lockdown. No difference in mortality was observed at any time. There were only seven adverse events directly related to COVID-19—all in the control arm.

Fortunately, participants were similarly compliant with taking daily pressure readings before and during lockdown. The study centres also made just as many contacts with participants. However, PA pressures were lower in both the monitored and control groups during lockdown, suggesting that patients with heart failure were generally more stable. What is the explanation for the fall in PA pressure? There was evidence of a reluctance to change medication, particularly renin–angiotensin inhibitors, mineralocorticoid receptor antagonists, or diuretics, during lockdown. This reluctance was most marked for increases in medication, which seems inconsistent with the fall in PA pressure. Why was there such reluctance to change doses of medicines? Perhaps patients were less enthusiastic about changes if they were worried about side effects that might trigger hospitalization. Perhaps the trial staff shared such concerns, particularly if the patient reported no change in symptoms. ‘Primum non nocere’ may have been the mantra during the COVID-19 restrictions. The likely explanation for the fall in PA pressures is a change in patient behaviour. Patients’ social interactions were markedly curtailed, which may have reduced dietary indiscretions and exposure to other respiratory viruses (such as influenza), and perhaps focused participants’ attention on health more generally, leading to better adherence to medication and lifestyle measures.

During lockdown, consistent with the fall in PA pressure, the rate of heart failure events dropped substantially in the control arm; hospitalizations fell by ∼20% and urgent attendance for intravenous diuretics almost halved. Similar reductions in hospitalizations associated with lockdowns have been reported for other trials and countries,^4,9^ which are thought to reflect re-prioritization of hospital services and a reluctance of both patients and healthcare professionals to risk exposing patients to nosocomial infection. GUIDE-HF provides an additional or alternative explanation for the reduction in hospitalizations; patients genuinely seem to have improved, with a change in behaviour leading to improved haemodynamics, which translated into a reduced risk of worsening heart failure. We need to find better ways of replicating this effect than another lockdown! However, with no difference in treatment or PA pressure between the two arms, it was hardly surprising that the trial found no difference in heart failure events during the COVID-19 lockdown. This does not preclude the possibility that monitoring triggered some hospitalizations for worsening congestion that might otherwise have been fatal. A more in-depth analysis might show an association between individual-patient haemodynamic response and the occurrence of events.

The evidence in GUIDE-HF collected pre-COVID (and probably generalizable to future, pandemic-free healthcare) is consistent with the previous CHAMPION trial,^10^ and several post-marketing studies in other geographies.^11,12^ At the end of February 2022, The FDA extended the indications for the CardioMEMS PA pressure monitor to include patients with heart failure ‘who have either been hospitalized for heart failure in the previous year and/or have elevated plasma concentrations of natriuretic peptides’, reflecting the inclusion criteria for GUIDE-HF. The pre-specified analysis and interpretation of the results provided by the investigators to mitigate the impact of COVID-19 on the GUIDE-HF trial have won the day, at least in the USA.

Ultimately, however, the most important message from GUIDE-HF is that we need more effective means for controlling congestion.^13,14^ Monitoring is futile unless it is followed by effective action. Trials targeting larger reductions in PA pressure should be conducted.

**Conflict of interest:** M.R.C. has provided consultancy advice to AstraZeneca, Abbott, Medtronic, Novartis, Servier, and Roche Diagnostics, and has received research funding for his organization from Abbott. J.G.F.C. has received personal honoraria for advisory boards and lectures from Abbott, Amgen, Astra-Zeneca Bayer, Bristol Myers Squibb, Johnson & Johnson Novartis, Medtronic, Myokardia, NI Medical, Pharmacosmos, Idorsia, Respicardia, Servier, Torrent, Vifor, and Viscardia, non-financial support from Boehringer-Ingelheim and Boston Scientific, and research funding for his institution from Bayer, Bristol Myers Squibb, Medtronic. and Vifor.
